# *δ*-Opioid Receptor Activation Inhibits Ferroptosis by Activating the Nrf2 Pathway in MPTP-Induced Parkinson Disease Models

**DOI:** 10.1155/2023/4130937

**Published:** 2023-02-10

**Authors:** Benchi Cai, Lifan Zhong, Yanhui Liu, Qian Xu, Tao Chen

**Affiliations:** Hainan General Hospital, Hainan Affiliated Hospital of Hainan Medical University, Haikou, Hainan 570311, China

## Abstract

**Introduction:**

Recent studies suggest the involvement of ferroptosis in the pathogenesis of Parkinson disease (PD). *δ*-Opioid receptors (DORs) have neuroprotective effects in PD. It is not known whether the neuroprotective effects of DORs in PD are attributable to the inhibition of ferroptosis. Therefore, we aimed to investigate the role of DORs in ferroptosis in MPTP-induced PD models.

**Methods:**

To identify the influence of DORs on ferroptosis in MPTP-induced PD models, we measured the malondialdehyde (MDA) and 4-hydroxynonenal (4-HNE) levels, analyzed the levels of ferroptosis-related proteins (GXP4 and SLC7a11) and Nrf2 expression by using western blotting, and assessed mitochondrial dysfunction by using JC-1 staining and transmission electron microscopy.

**Results:**

DOR activation reduced the 4-HNE and MDA levels, increased the GXP4 and SLC7a11 levels, and ameliorated mitochondrial dysfunction in MPTP-induced PD models. These neuroprotective effects of DORs could be blocked by Nrf2-siRNA. Thus, the effects of DORs on ferroptosis in PD models were partially controlled by Nrf2, which regulated GXP4 and SLC7a11 synthesis.

**Conclusion:**

DORs exert neuroprotective effects in PD models by inhibiting ferroptosis partially via activating the Nrf2 pathway.

## 1. Introduction

Parkinson disease (PD) is the second most common neurodegenerative disease in the world [1]. PD predominantly occurs in elderly patients. The clinical manifestations of PD include both motor and nonmotor symptoms that gradually progress over the course of the disease and ultimately lead to the loss of self-care ability, which increases family and social burden. Although it is known that the main pathological change in PD is the loss of dopaminergic neurons in the substantia nigra and striatum, the precise mechanism underlying this change is not yet clear. The cause of this neuronal loss is closely related to the process of cell death, specifically, ferroptosis and other types of programmed cell death, which have now become a focus of PD research [2].

Ferroptosis [3] is an iron-dependent, erastin-specific-induced programmed cell death characterized by changes such as shrinkage of mitochondria, loss of mitochondrial crests, exhaustion of reduced glutathione (GSH), and accumulation of reactive oxygen species (ROS). Inhibitors of programmed cell death do not inhibit ferroptosis, though some iron chelators do reverse it. The key to ferroptosis is GSH. Under the catalytic action of glutathione peroxidase 4 (GPX4), GSH can help prevent the abnormal accumulation of cellular ROS. The targeted inhibition of GPX4 leads to the accumulation of lipid peroxides and induces ferroptosis [4]. Studies indicate that ferroptosis is an early and characteristic type of cell death in PD [5–8]. Dj-1, a protein linked to early-onset PD, inhibits ferroptosis [9]. An iron-chelating agent was shown to exert a significant protective effect against PD in mice [10]. The previous findings offer new research avenues to develop strategies for the early treatment of PD.

Opiates are a group of diverse neurotransmitters that include endorphins and enkephalins. Opioid receptors also exhibit multiple polymorphisms (e.g., *μ*, *δ*, and *κ* receptors) and are widely distributed in the central nervous system. Delta opioid receptors (DORs) are mainly distributed in the striatum. DORs play an important neuroprotective role [11, 12]. Our previous studies showed that the activation of DORs increases the phosphorylation of cAMP response element-binding (CREB) protein, stabilizes mitochondrial membrane potential [13], and weakens the overexpression/aggregation of synuclein [14], which plays a neuroprotective role in PD models. The functions of opioid receptors are yet to be fully characterized.

Nrf2 protein is a leucine zipper transcription factor that regulates the expression of multiple genes and serves a variety of physiological functions. Studies have shown that the activation of opioid receptors can activate the Nrf2 pathway [15–19] and that Nrf2 is a key transcriptional regulator of ferroptosis [20, 21]. Therefore, we hypothesized that the activation of DORs exerts a neuroprotective effect by activating Nrf2 to inhibit ferroptosis in PD models. Thus, the aim of the present study is to further explore this potential mechanism underlying the neuroprotective effect of DORs and provide a theoretical basis for the development of new anti-PD drugs.

## 2. Materials and Methods

### 2.1. Materials

For cell cultures, we obtained RPMI 1640 medium and fetal bovine serum (FBS) from Sigma-Aldrich (St. Louis, MO, USA). [d-Ala^2^, d-Leu^5^]-Enkephalin (DADLE), a specific DOR agonist; naltrindole, a specific DOR antagonist and ferrostatin-1, a ferroptosis inhibitor, were purchased from MedChemExpress Co. (Shanghai, China). The antibodies for western blot analysis were purchased from Abcam Co. (St. Louis, MO, USA): antibeta actin antibody, anti-Nrf2 antibody, anti-GPX4 antibody, and anti-xCT antibody.

### 2.2. Transfection

PC12 cells were transfected with anti-Nrf2 short interfering RNA (siRNA). Scrambled siRNA was used as a control. The siRNAs were obtained from Obio Technology (Shanghai, China). The siRNA sequences were as follows: Nrf2-siRNA (sense), UGACAGAAGUUGACAAUUAdTdT; Nrf2-siRNA (antisense), UAAUUGUCAACUUCUGUCAdTd; scrambled-siRNA (sense), UUCUCCGAACGUGUCACGUdTdT; scrambled-siRNA (antisense), ACGUGACACGUUCGGAGAAdTdT. Transient transfection was accomplished using Lipofectamine 3000 reagent (ThermoFisher Scientific, Uppsala, Sweden) according to the manufacturer's instructions.

### 2.3. Cell Culture

PC12 cells (ATCC, Manassas, VA, USA) were grown in RPMI 1640 medium supplemented with 10% FBS and 1% penicillin-streptomycin in a humidified incubator containing 5% CO_2_ at 37°C. For establishment of the PD model, the PC12 cells were treatment of MPTP (1 mmol/l, 24 h). The cells (1 × 10^5^ cells/mL) were placed in 60-mm dishes for western blot analysis and mitochondrial membrane potential assay and in 96-well plates for cell viability and cytotoxicity assays.

### 2.4. Mouse Models

Male C57BL/6 mice were obtained from the Animal House of Hainan Medical University. We randomly divided 60 C57BL/6 mice among the following groups: control, MPTP, MPTP + ferrostatin-1, MPTP + DADLE, MPTP + naltrindole, and MPTP + DADLE + naltrindole group. For establishment of the PD model, the mice were administered abdominal injections of MPTP (1-methyl-4-phenyl-1,2,3,6-tetrahydropyridine) at a dosage of 30 mg/kg once per day for 5 days. The control mice were given the same volume of phosphate-buffered saline (PBS). DADLE (10 mg/kg) and naltrindole (10 mg/kg) were intraperitoneally injected once per day for 7 days. In the MPTP + DADLE + naltrindole group, DADLE and naltrindole were injected 30 min apart. All animal experimental procedures were approved by the Laboratory Animal Ethics Committee of Hainan Medical University.

### 2.5. Open Field Test

This approach has been used by our research team before [22]. Each mouse was placed in the center of the open-field apparatus. The center zone was defined as a square, 10 cm away from the walls. The distance traveled and time spent in the center zone by each animal was recorded for 10 min by using a video-imaging system (EthoVisionXT; Noldus Information Technology, Wageningen, The Netherlands).

### 2.6. Cytotoxicity and Cell Viability Assessments

Cytotoxicity was quantified using lactate dehydrogenase (LDH) activity measurement kits (KeyGEN BioTECH, Nanjing, China) according to the manufacturer's instructions. Cell viability was assessed using CCK-8 kits (TransGen Biotech, Beijing, China) according to the manufacturer's instructions. The absorbance value (optical density, 450 nm) was measured using a microplate reader (BioTek Instrumentals Inc., Winooski, VT, USA).

### 2.7. Immunofluorescence

For immunofluorescence analysis, tissue samples from the substantia nigra region were fixed with 4% paraformaldehyde for 20 min, permeabilized with 0.3% Triton X-100 in PBS for 30 min, and incubated with 10% bovine serum albumin for 1 h at room temperature. Primary antibodies were added, and the incubation was continued at 4°C overnight. The sections were then washed with PBS and incubated with fluorescent labeled secondary antibodies (mouse monoclonal antityrosine hydroxylase antibody, 1 : 2000; Sigma-Aldrich). Finally, the sections were examined using confocal microscopy (TCS SP8; Leica, Solms, Germany). Immunofluorescence was quantified using ImageJ software.

### 2.8. Lipid Peroxidation Measurement

To evaluate ferroptosis, we measured the cellular malondialdehyde (MDA) and 4-hydroxynonenal (4-HNE) concentrations in the various study groups by using the Lipid Peroxidation (MDA) Assay kit (Sigma-Aldrich) and Lipid Peroxidation (4-HNE) Assay kit (Abcam), respectively, according to the manufacturers' instructions.

### 2.9. Western Blot Analysis

Samples from PC12 cells or the mouse models were separated using 10% sodium dodecyl sulfate polyacrylamide gel electrophoresis under denaturing conditions and then transferred to polyvinylidene difluoride membranes. Nonspecific binding was blocked using a blocking buffer. The membranes were then incubated overnight at 4°C with specific primary antibodies diluted in Tris-buffered saline supplemented with 0.1% Tween20 (TBST) and 1% nonfat dry milk. After being washed 3 times with TBST, the membranes were incubated for 1 h at room temperature with goat antirabbit IgG diluted to a ratio of 1 : 5000 in 1% nonfat dry milk. The membranes were again washed 3 times with TBST, and immunoreactive bands were observed using Pierce® ECL Western Blotting Substrate (ThermoFisher Scientific, Uppsala, Sweden). For quantitative analysis of proteins, the membranes were stripped using Restore™ Western Blot Stripping Buffer (ThermoFisher Scientific, Uppsala, Sweden) and reprobed with anti-*β*-actin antibody (1 : 5000).

### 2.10. Mitochondrial Membrane Potential Measurement

The mitochondrial membrane potential (ΔΨ*m*) of PC12 cells was measured using fluorescence microscopy and the JC-1 Mitochondrial Membrane Potential Assay Kit (Abcam Co., St. Louis, MO, USA) according to the manufacturers' instructions. Healthy cells containing JC-1 aggregates were identified under a fluorescence microscope at excitation and emission wavelengths of 540 and 570 nm, respectively. Unhealthy cells containing JC-1 monomers were identified by detecting fluorescein isothiocyanate under a fluorescence microscope at excitation and emission wavelengths of 485 and 535 nm, respectively.

### 2.11. Transmission Electron Microscopy

Substantia nigra samples from the mice were cut into 60–80-nm-thin sections on an ultramicrotome and imaged using a transmission electron microscope (JEM-1400plus; JEOL, Tokyo, Japan).

### 2.12. Statistical Analysis

All values were presented as the mean ± standard error. One-way analysis of variance followed by the Bonferroni test was used to conduct multiple pairwise comparisons to identify significant differences. *P* < 0.05 was deemed to indicate statistical significance.

## 3. Results

### 3.1. Neuroprotective Effects of DORs in MPTP-Induced PD Models

**Figure 1:**
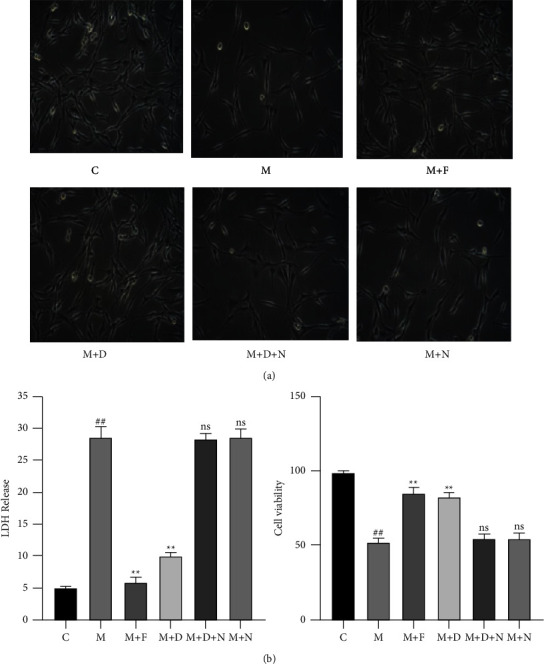
Neuroprotective effects of DORs in MPTP-treated PC12 cells. (a) PC12 cells subjected to different treatments were observed and photographed using a microscope (200×). (b) Cytotoxicity was evaluated by measuring LDH release; the CCK-8 assay was used to evaluate cell viability. Data are presented as the mean ± S.E. of three independent experiments. ^##^*P* < 0.01 vs. the control group; ^*∗∗*^*P* < 0.01 and ns, *P* > 0.05 vs. the MPTP-treated group. DOR, *δ*-opioid receptor; MPTP, 1-methyl-4-phenyl-1,2,3,6-tetrahydropyridine; LDH, lactate dehydrogenase; C, control; M, MPTP; F, ferrostatin-1; D, [D-Ala^2^, D-Leu^5^]-enkephalin (DADLE); N, naltrindole; ns, not significant.

MPTP-treated PC12 cells were further treated with different concentrations of DADLE (0, 0.1, 1, 10, and 100 nM) for 24 h. CCK-8 and LDH assays revealed that the strongest effects were observed in cells treated with 10 nM DADLE or 10 nM naltrindole (Supplementary Figures S1A and S1B). Next, MPTP-treated PC12 cells were treated with 10 nM DADLE and/or 10 nM naltrindole for 24 h and then observed and photographed using an inverted microscope. The PC12 cells in the normal control group, DADLE + MPTP group, and ferrostatin-1 + MPTP group appeared normal (Figure 1(a)). In contrast, freely floating cells were observed in the MPTP, DADLE + naltrindole + MPTP, and naltrindole + MPTP groups (Figure 1(a)). The CCK-8 assay showed that compared with the control group, the MPTP group showed a significant decline in cell viability (about 50%; Figure 1(b)). Compared with the MPTP group, the MPTP + ferrostatin-1 group and MPTP + DADLE group showed increased cell viability (32.8% ± 1.797%, *P* < 0.05 and 30.00% ± 1.643%, *P* < 0.05). The LDH assay showed that ferrostatin-1 (22.70% ± 0.6682%, *P* < 0.05) and DADLE (18.72% ± 0.6655%, *P* < 0.05) significantly decreased the rate of LDH leakage from MPTP-treated PC12 cells (Figure 1(b)). These results indicated that treatment with DADLE and/or ferrostatin-1 effectively enhanced the viability of MPTP-treated PC12 cells.

**Figure 2:**
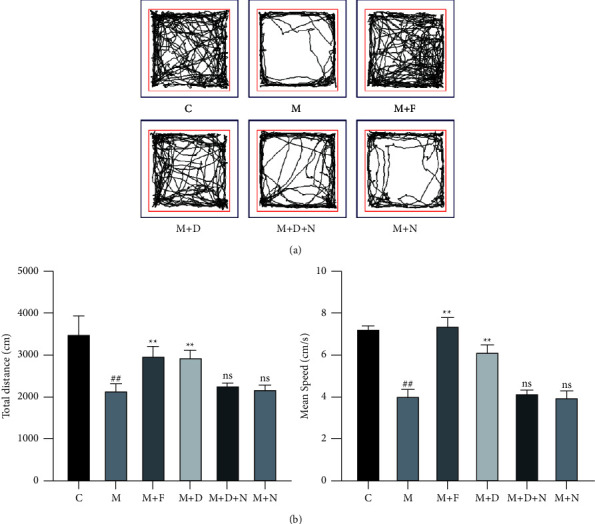
Neuroprotective effects of DORs in mice with MPTP-induced PD. (a, b) Open field test, analysis of total distance and mean speed. Data are presented as the mean ± S.E. of three independent experiments. ^##^*P* < 0.01 vs. the control group; ^*∗∗*^*P* < 0.01 and ns, *P* > 0.05 vs. the MPTP-treated group. DOR, *δ*-opioid receptor; MPTP, 1-methyl-4-phenyl-1,2,3,6-tetrahydropyridine; PD, Parkinson disease; DADLE, [D-Ala^2^, D-Leu^5^]-enkephalin; ns, not significant.

Next, we assessed the role of DORs in the mouse model of MPTP-induced PD by conducting an open field test. After 1 week of treatment with DADLE, the movement distance and mean speed of the MPTP + DADLE-treated mice were significantly greater than those of the MPTP-treated mice (*P* < 0.01, Figures 2(a) and 2(b). Similar results were obtained for ferrostatin-1. These findings suggest that DADLE and ferrostatin-1 aided the recovery of motor activity in mice with MPTP-induced PD.

**Figure 3:**
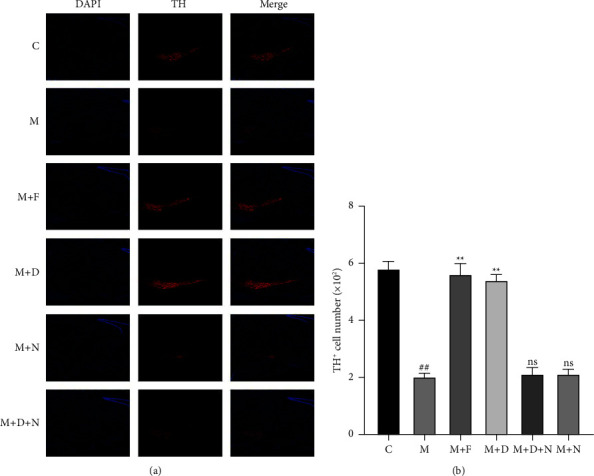
DOR reduced MPTP-induced dopaminergic neuronal degeneration in substantia nigra. (a) Representative immunofluorescence images of TH-positive cells in the SN of the four groups; (b) quantitative analysis of TH-positive regions in the SN in the groups (*n* = 5). The quantitative analysis of the TH-positive regions was performed using ImageJ. All statistical differences were tested using a *t*-test. Data are expressed as the mean ± SEM (*n* = 5/group). The significance is expressed as ^##^*P* < 0.01 vs. control group. ^*∗∗*^*P* < 0.01 vs. MPTP group. ns, not significant vs. MPTP group. DOR, *δ*-opioid receptor; MPTP, 1-methyl-4-phenyl-1,2,3,6-tetrahydropyridine; PD, Parkinson disease; DADLE, [d-Ala^2^, d-Leu^5^]-enkephalin; ns, not significant.

**Figure 4:**
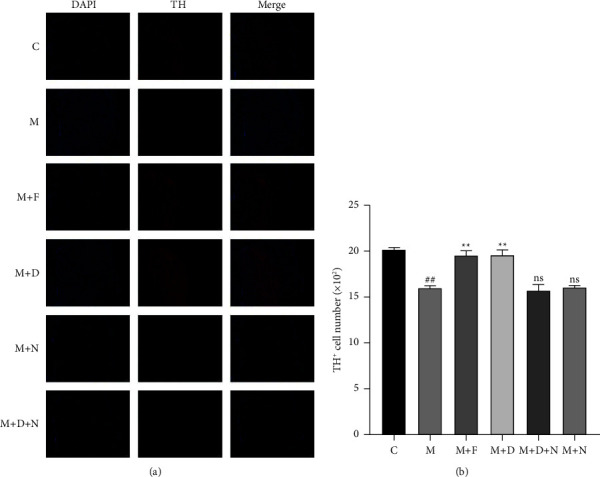
DOR reduced MPTP-induced dopaminergic neuronal degeneration in striatum. (a) Representative immunofluorescence images of TH-positive cells in the striatum of the four groups; (b) quantitative analysis of TH-positive regions in the striatum in the groups (*n* = 5). The quantitative analysis of the TH-positive regions was performed using ImageJ. All statistical differences were tested using a *t*-test. Data are expressed as the mean ± SEM (*n* = 5/group). The significance is expressed as ^##^*P* < 0.01 vs. control group. ^*∗∗*^*P* < 0.01 vs. MPTP group. ns, not significant vs. MPTP group. DOR, *δ*-opioid receptor; MPTP, 1-methyl-4-phenyl-1,2,3,6-tetrahydropyridine; PD, Parkinson disease; DADLE, [d-Ala^2^, d-Leu^5^]-enkephalin; ns, not significant.

To determine whether DORs help protect against MPTP-induced damage to nigrostriatal dopaminergic neurons, we performed immunofluorescence analysis of tyrosine hydroxylase, a marker for dopaminergic neurons, in tissue samples of the substantia nigra and striatum region collected from mice with PD. The results showed that DADLE and ferrostatin-1 decreased the MPTP-induced loss of dopaminergic neurons and nerve fibers in the substantia nigra (Figures 3 and 4).

### 3.2. DOR Activation Inhibits Ferroptosis in MPTP-Induced PD Models

**Figure 5:**
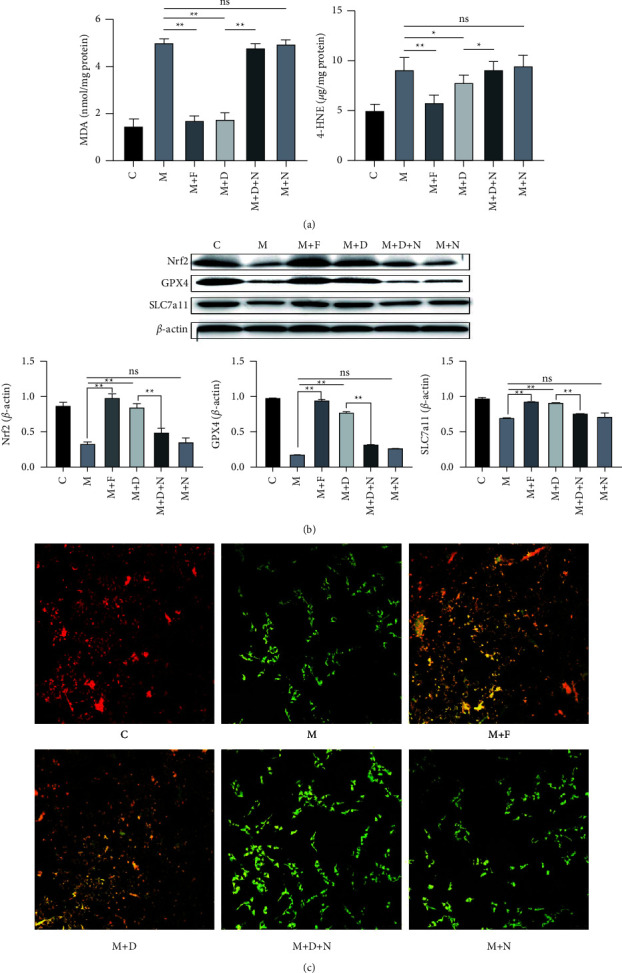
DOR activation inhibits ferroptosis in MPTP-treated PC12 cells. (a) MDA and 4-HNE levels in PC12 cells after different treatments, as detected using the MDA and 4-HNE assay kits. (b) Representative western blots of Nrf2, GPX4, and SLC7a11, and quantitative analysis of the protein levels of Nrf2, GPX4, and SLC7a11. (c) Mitochondrial membrane potential as assessed using JC-1 staining of PC12 cells subjected to different treatments (scale bar = 50 *μ*m). Data are presented as the mean ± S.E. of three independent experiments. ^*∗*^*P* < 0.05; ^*∗∗*^*P* < 0.01; ns, *P* > 0.05. DOR, DOR, *δ*-opioid receptor; MPTP, 1-methyl-4-phenyl-1,2,3,6-tetrahydropyridine; MDA, malondialdehyde; 4-HNE, 4-hydroxynonenal; GPX4, glutathione peroxidase 4; DADLE, [D-Ala^2^, D-Leu^5^]-enkephalin; ns, not significant; C, control; M, MPTP; F, ferrostatin-1; D, DADLE; N, naltrindole.

**Figure 6:**
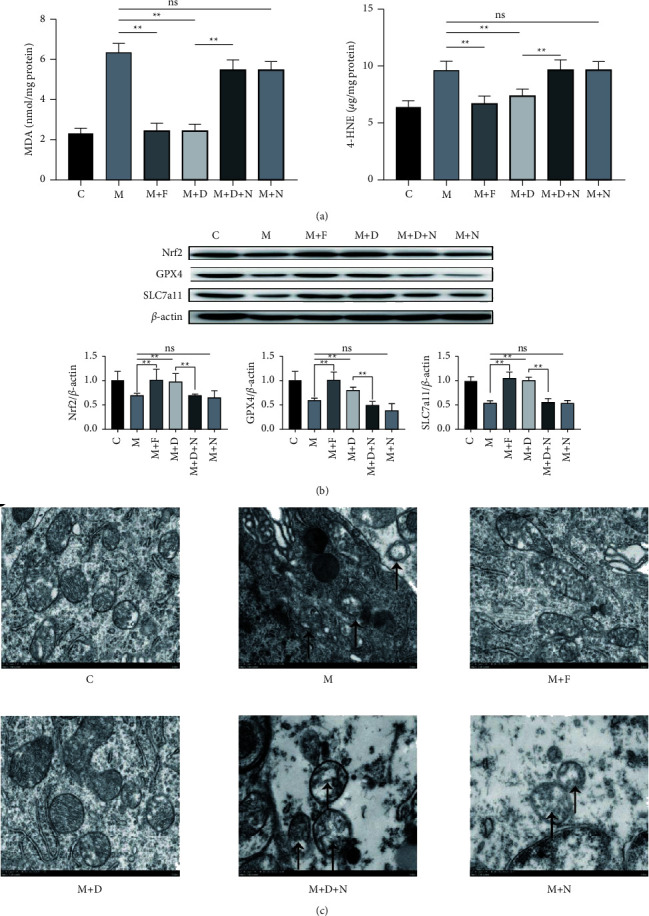
DOR activation inhibits ferroptosis in the MPTP-induced mouse model of PD. (a) MDA and 4-HNE levels in the substantia nigra of mice after different treatments, as detected using MDA and 4-HNE assays kits. (b) Representative western blots of Nrf2, GPX4, and SLC7a11, and quantitative analysis of the protein levels of Nrf2, GPX4, and SLC7a11. (c) Mitochondrial substructure was observed using transmission electron microscopy in mice subjected to different treatments (scale bar = 500 nm). ↑, mitochondrial membrane rupture and loss of crista. Data are presented as the mean ± S.E. of three independent experiments. ^*∗∗*^*P* < 0.01; ns, *P* > 0.05. DOR, *δ*-opioid receptor; MPTP, 1-methyl-4-phenyl-1,2,3,6-tetrahydropyridine; PD, Parkinson disease; MDA, malondialdehyde; 4-HNE, 4-hydroxynonenal; GPX4, glutathione peroxidase 4; DADLE, [D-Ala^2^, D-Leu^5^]-enkephalin; ns, not significant; C, control; M, MPTP; F, ferrostatin-1; D, DADLE; N, naltrindole.

Ferroptosis may be an early pathological change in PD, and increased lipid peroxide content is an important marker of ferroptosis. GPX4 and SLC7a11 are key proteins that regulate ferroptosis, and mitochondrial dysfunction plays a key role in ferroptosis. Therefore, after the activation of DORs in MPTP-induced PD models, we measured the MDA and 4-HNE contents, the protein expression levels of GPX4 and SLC7a11, and mitochondrial dysfunction. We found that MPTP induced MDA and 4-HNE accumulation induced mitochondrial dysfunction and decreased the protein expressions of GPX4 and SLC7a11, all of which indicated that ferroptosis is involved in PD (Figures 5 and 6). Treatment of PC12 cells and PD mouse models with ferrostatin-1 or DADLE remarkably reduced the accumulation of MDA and 4-HNE (Figures 5(a) and 6(a)), decreased the protein expressions of GPX4 and SLC7a11 (Figures 5(b) and 6(b)), and restored the mitochondrial membrane potential (Figure 5(c), Supplementary Figure S3A). In addition, transmission electron microscopy showed that the mitochondrial membranes appeared intact, and the mitochondrial cristae were restored after the DADLE and ferrostatin-1 treatments (Figure 6(c)). Collectively, the previous results show that the activation of DORs had an inhibitory effect on ferroptosis in MPTP-treated PC12 cells and mice.

### 3.3. DOR Activation Enhances Nrf2 Protein Expression in MPTP-Induced PD Models

As Nrf2 is a crucial factor regulating ferroptosis, we next investigated whether the activation of DORs by DADLE or ferrostatin-1 could increase the expression of Nrf2. Western blot analysis revealed that after treatment with DADLE or ferrostatin-1, Nrf2 expression was upregulated in PC12 cells and PD mouse models (Figures 5(b) and 6(b)), which demonstrated that DORs increased Nrf2 synthesis in MPTP-induced PD models.

### 3.4. DORs Regulate Ferroptosis via the Nrf2 Pathway in MPTP-Treated PC12 Cells

**Figure 7:**
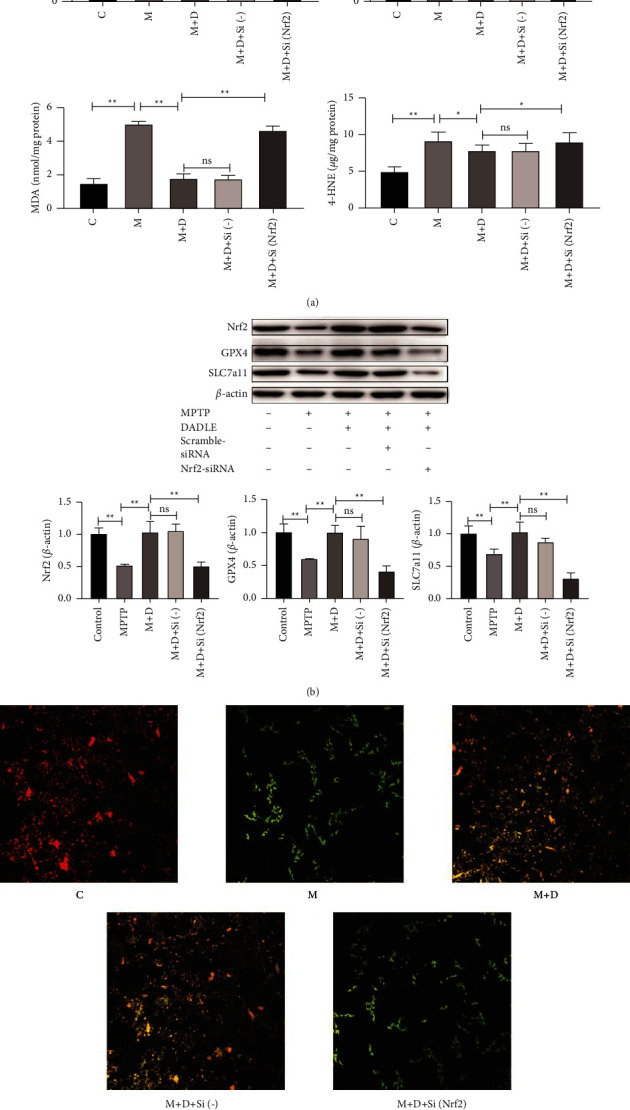
DORs regulate ferroptosis via the Nrf2 pathway in the MPTP-treated PC12 cells. (a) LDH-release assay, CCK-8 assay, and measurement of lipid peroxidation. (b) Representative western blots of Nrf2, GPX4, and SLC7a11, and quantitative analysis of the protein levels of Nrf2, GPX4, and SLC7a11. (c) Mitochondrial membrane potential was assessed using JC-1 staining of PC12 cells subjected to different treatments (scale bar = 50 *μ*m). Data are presented as the mean ± S.E. of three independent experiments. ^*∗*^*P* < 0.05; ^*∗∗*^*P* < 0.01; ns, *P* > 0.05. DOR, *δ*-opioid receptor; MPTP, 1-methyl-4-phenyl-1,2,3,6-tetrahydropyridine; LDH, lactate dehydrogenase; GPX4, glutathione peroxidase 4; DADLE, [D-Ala^2^, D-Leu^5^]-enkephalin; ns, not significant; C, control; M, MPTP; F, ferrostatin-1; D, DADLE; N, naltrindole.

**Figure 8:**
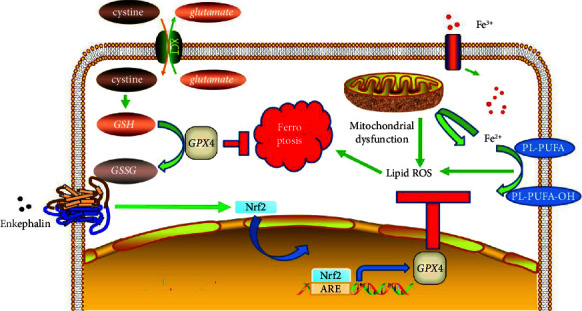
The antiferroptosis mechanism of DORs. DOR, *δ*-opioid receptor; GPX4, glutathione peroxidase 4; GSH, reduced glutathione; GSSG, oxidized glutathione; PL-PUFA, phospholipid-bound polyunsaturated fatty acid; ROS, reactive oxygen species.

To further analyze the neuroprotective and antiferroptosis effects of DOR, we transfected PC12 cells with anti-Nrf2 siRNA. We found that in PC12-siNrf2 cells, Nrf2 expression was remarkably reduced to 42.16% ± 4.44% of the expression in PC12 cells (*P* < 0.05; Supplementary Figure S2). Furthermore, the neuroprotective and antiferroptosis effects of DORs were preserved in MPTP-treatedPC12-scrambled siRNA cells but drastically diminished in MPTP-treatedPC12-siNrf2 cells (Figure 7, Supplementary Figure S3B). Collectively, these findings show that DORs protect PC12 cells from MPTP-induced ferroptosis via the promotion of Nrf2 expression (Figure 8).

## 4. Discussion

The current treatment for PD consists of increasing the neurotransmission of dopamine to provide symptomatic relief. Although dopaminergic drug therapy can delay the progression of the disease and improve symptoms, a high concentration of dopamine may have neurotoxic effects. A specific dopaminergic neuroprotective drug is still lacking [23]. A better understanding of disease-related pathological mechanisms and their relationship with the cell death processes driving dopaminergic neuronal loss is required for the discovery of novel therapeutic targets for the treatment of PD.

PD-associated neuronal cell death was previously attributed to apoptosis. Recently, however, ferroptosis was reported to be a possible mechanism of neuronal cell death in PD [8]. Some pathological features of PD, such as iron overload, increased lipid peroxidation, decreased GSH levels, DJ-1 consumption, and decreased coenzyme Q10, are known to be involved in ferroptosis, which strongly implicates this programmed cell death process in PD [7, 24, 25]. Although MPTP-induced death and ferroptosis shared some features, such as occurrence of lipid peroxidation and inhibition by Fer-1, MPTP-induced death seemed to be distinct from ferroptosis because MPTP-induced death was accompanied by ATP depletion and mitochondrial swelling [26]. Further investigation of other indirect regulated factors may be useful for understanding the mechanisms of neuronal loss and for treatment of Parkinson's disease.

Ferroptosis is a type of programmed cell death that is activated when iron-dependent lipid peroxidation reaches lethal levels, and the only unique morphological feature is wrinkling of the mitochondrial membrane. The peroxidation substrates in ferroptosis are phospholipid-bound polyunsaturated fatty acids in the cell membrane; the lipid peroxides decompose into toxic derivatives such as 4-HNE and MDA [27]. GPX4 is a key enzyme in the ferroptosis pathway [28]. GPX family members such as GPX1–GPX8 have been found in mammals [29]. Direct GPX4 inactivation by RAS-selective lethal 3 (RSL3) is commonly used to induce ferroptosis under experimental conditions. GPX4 provides electrons for prototype glutathione (GSH), releasing oxidized glutathione [30]. GSH is synthesized in cells from glutamate and cysteine, of which the latter is the rate-limiting substrate. Cysteine is synthesized from methionine through the transsulfuration pathway or absorbed as an oxidized cystine dimer by the XcT (encoded by SLC7A11) reverse transporter, which is required for intracellular cysteine transport. XcT also plays an important role in regulating ferroptosis [31]. It is generally accepted that the levels of GPX4, MDA, 4-HNE, and SLC7A11 serve as powerful indices of the level of ferroptosis.

Several studies have demonstrated the neuroprotective functions of DORs [11, 32–36]. In cultured cortical neurons, DOR activation significantly reduces glutamate-induced neurotoxicity [37], and the neuroprotective effects of DORs are mediated through the regulation of ion channels [38, 39]. Some studies have suggested that opioid receptor agonists can be used as dopamine alternatives in the treatment of PD [40]. DOR agonists, such as DADLE and UFP-512, have displayed the ability to delay neuronal death in PD [13, 35, 41–44]. However, the mechanism underlying the neuroprotective effects of DORs is not completely understood. Some studies have shown that DORs can promote the expression and nuclear translocation of Nrf2 [19]. Nrf2 is the main transcription factor regulating antioxidant reactions and has an antiferroptosis effect [20, 21]. In the presence of oxidative stress, Nrf2 is translocated to the nucleus, which induces the expression of endogenous antioxidant proteins that prevent lipid peroxidation [45]. Persistent exposure to erastin results in the upregulation of the Nrf2-dependent enzyme cystathionine-*β*-synthase, which is responsible for the biosynthesis of cysteine, which fights cell death [46]. Nrf2 also controls NAD(P)H, several iron-metabolizing proteins, GPX4, and other key proteins for the biosynthesis of GSH (e.g., XcT and glutathione synthetase). The oxidative stress-induced Nrf2 response is not specific to ferroptosis, as Nrf2 inhibition is also associated with apoptotic cell death. Thus, the role of Nrf2 in PD may be related to multiple types of cell death.

In summary, we have explored the neuroprotective and antiferroptosis effects of DORs in PD models and clarified the mechanism underlying these effects of DORs (Figure 8). The main findings of our study are as follows: (1) DORs confer neuroprotection in MPTP-induced PD models, (2) DOR activation inhibits ferroptosis in MPTP-induced PD models, (3) DOR activation enhances Nrf2 protein expression in MPTP-induced PD models, and (4) DORs regulate ferroptosis via the Nrf2 pathway in MPTP-treated PC12 cells.

Our study findings indicate that DORs suppress the decline in cell viability and mitigate ferroptosis in MPTP-induced PD by activating the Nrf2 pathway. Thus, DOR-based therapy and antiferroptosis agents represent novel therapeutic approaches that might be developed for the treatment of PD. In addition, we look forward to the DOR exerting a successful therapeutic effect and might be administered either as an adjuvant in combination with other therapeutic strategies. Especially, the combined utilization of the DOR-based therapy with other traditional treatment such as supplement of dopamine. In future studies, our results can be verified in Nrf2 knockout mice, and the mechanism underlying the neuroprotective effects of DORs can be further investigated. In addition, our investigation sought to elucidate the mechanisms of Nrf2 alteration, especially in nucleus and cytoplasm separately.

## Data Availability

The dates of this study are stored at the Hainan Medical University. The datasets supporting the conclusion of this study are available from the corresponding author on reasonable request.

## Supplementary Materials

Supplementary Materials The online version of this article contains supplementary material, which is available for authorized users. Supplementary Figure S1: Screening of DADLE and naltrindole concentrations in PC12 cells. Supplementary Figure S2: PC12 cells transfected with siRNA targeting Nrf2. Supplementary Figure S3: Quantitative analysis of JC-1.
