# Supplementary material for: δ-Opioid Receptor Activation Inhibits Ferroptosis by Activating the Nrf2 Pathway in MPTP-Induced Parkinson Disease Models
Source: Evid Based Complement Alternat Med. 2023 Feb 10;2023:4130937. doi: 10.1155/2023/4130937 (PMC9937764; doi:10.1155/2023/4130937)
Supplement: Supplementary Materials — The online version of this article contains supplementary material, which is available for authorized users. Supplementary Figure S1: Screening of DADLE and naltrindole concentrations in PC12 cells. Supplementary Figure S2: PC12 cells transfected with siRNA targeting Nrf2. Supplementary Figure S3: Quantitative analysis of JC-1. [file 4130937.f1.docx]

**Supplementary Figure legends**

**
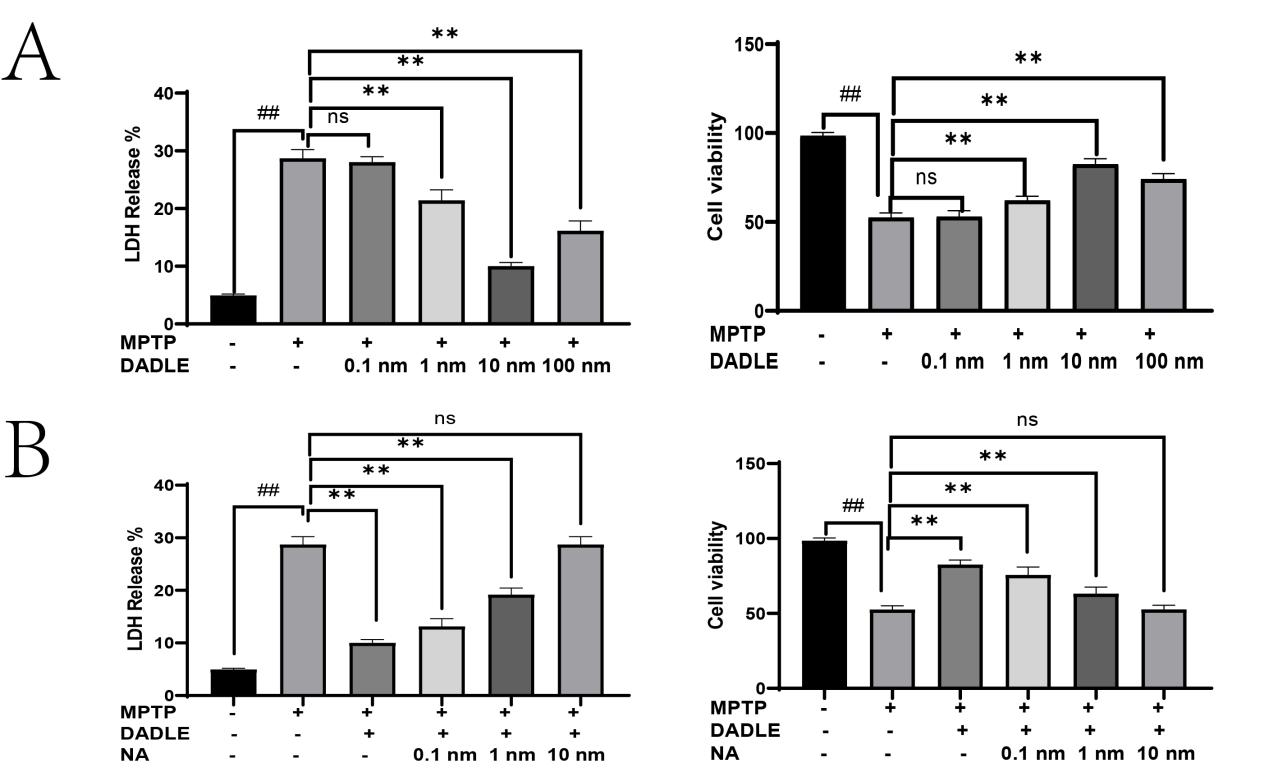
**

**Supplementary Figure S1.** Screening of DADLE and naltrindole concentrations in PC12 cells. (A) Screening of DADLE concentrations in PC12 cells. (B) Screening of naltrindole concentrations in PC12 cells. ##*P* < 0.01 *vs.* the control group; ***P* < 0.01 and ns, *P* > 0.05 *vs.* the MPTP-treated group. LDH, lactate dehydrogenase; MPTP, 1-methyl-4-phenyl-1,2,3,6-tetrahydropyridine; DADLE, [d-Ala^2^, d-Leu^5^]-enkephalin; NA, naltrindole; ns, not significant


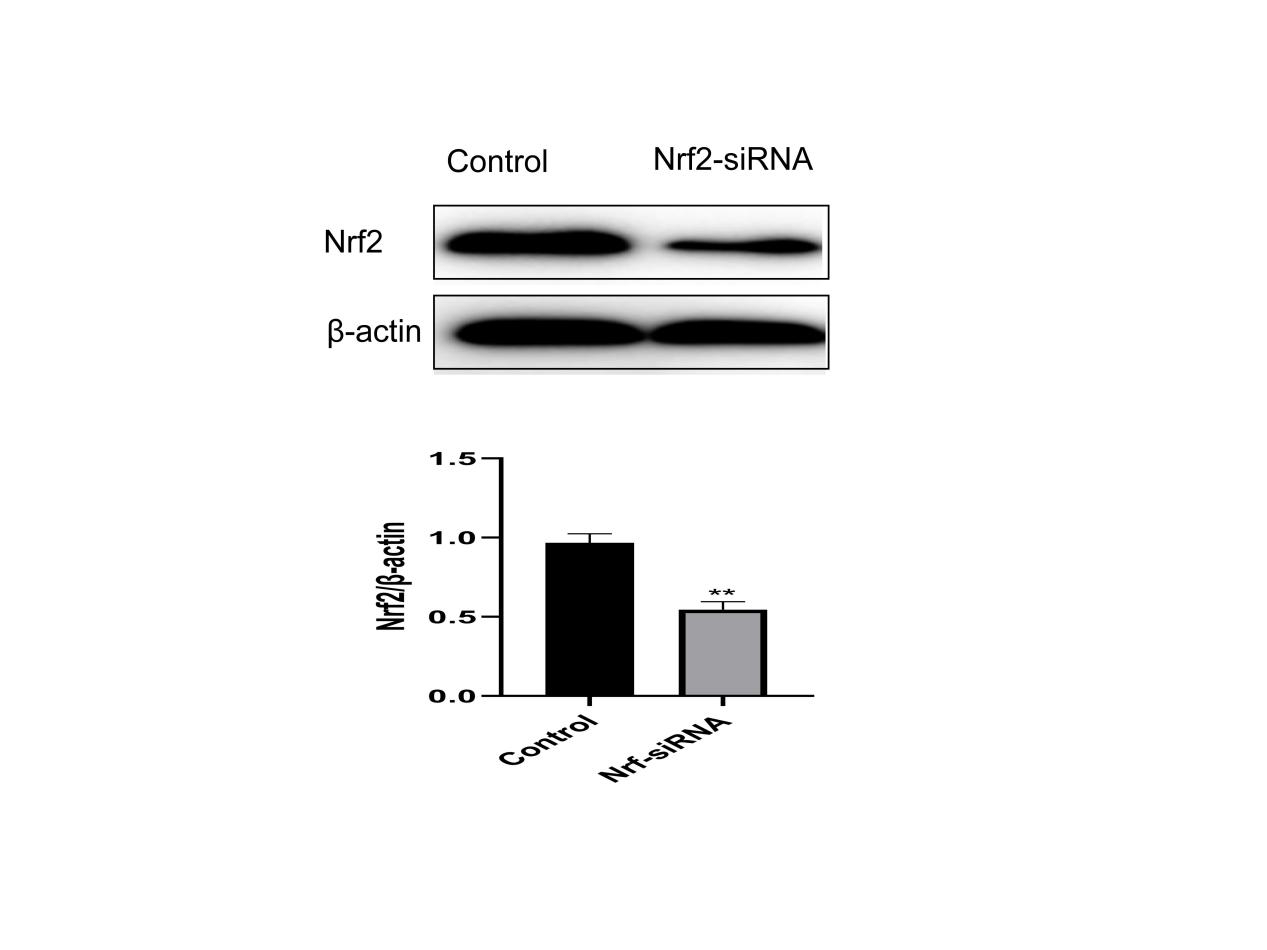


**Supplementary Figure S2.** PC12 cells transfected with siRNA targeting Nrf2. ***P* < 0.01 *vs.* the control group


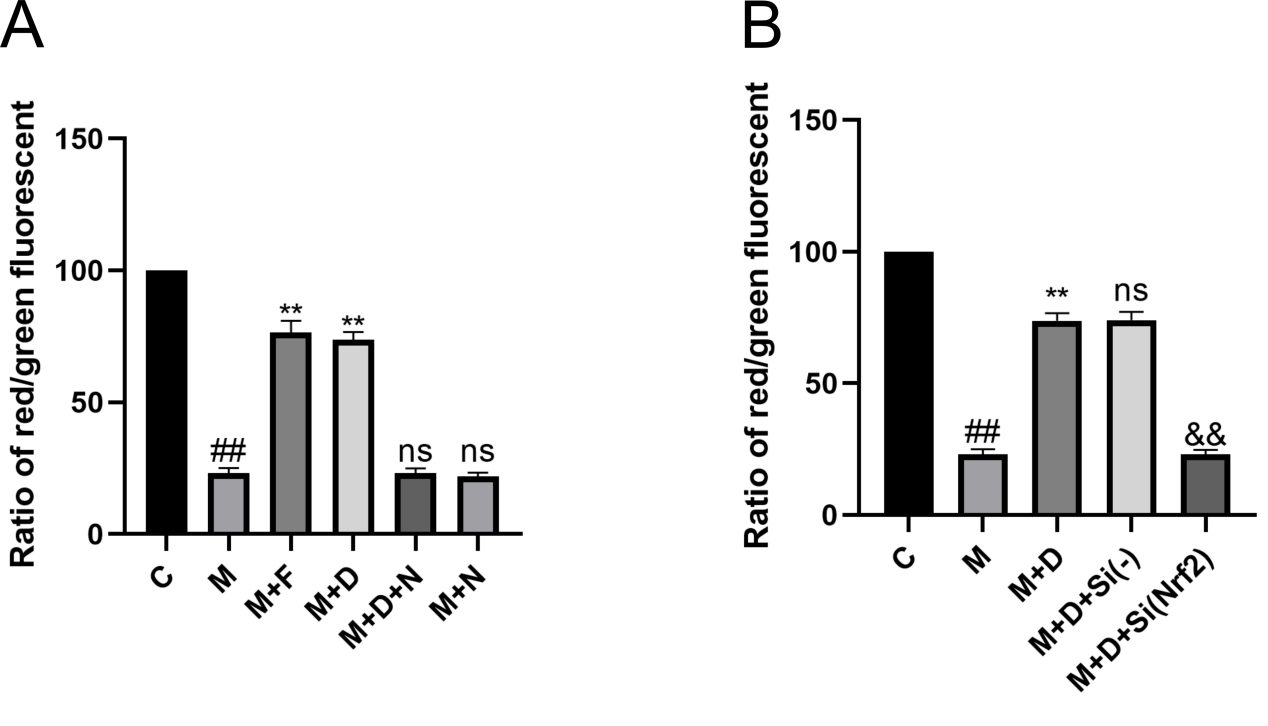


**Supplementary Figure S3.** Quantitative analysis of JC-1. (A) Quantitative analysis of JC-1 of figure 4C.##*P* < 0.01 *vs.* the control group; ***P* < 0.01 and ns, *P* > 0.05 *vs.* the MPTP-treated group; (B) Quantitative analysis of JC-1 of figure 6C. ##*P* < 0.01 *vs.* the control group; ***P* < 0.01 *vs.* the MPTP-treated group. &&*P* < 0.01 and ns, *P* > 0.05 *vs.* the MPTP+DADLE group
